# Quality of care for patients with ST-segment Elevation Myocardial Infarction before COVID-19 pandemic in Cuba: review of 17 reports with 7823 patients

**DOI:** 10.31053/1853.0605.v80.n4.42094

**Published:** 2023-12-26

**Authors:** Luis E Remedios-Carbonell, Dayani Arteaga-Guerra, Maidelis Prieto-Guerra, Geovedys Martinez-Garcia, Maikel Santos-Medina, Miguel A Rodríguez-Ramos

**Affiliations:** 1 Cardiology Department, General Hospital Camilo Cienfuegos Sancti-Spiritus Cuba; 2 Emergency Depratment, General Hospital Camilo Cienfuegos Sancti-Spiritus Cuba

**Keywords:** calidad de atención, infarto agudo de miocardio, registros médicos, quality of healthcare, acute myocardial infarction, medical records, qualidade dos cuidados de saúde, infarto agudo do miocárdio, registros médicos

## Abstract

Background: In Cuba, there is neither a registry of ST Elevation Myocardial Infarction (STEMI), nor are analysis of performance measures widely reported.Objective: A review of Cuban studies of patients with STEMI was carried out to describe quality of medical care.Methods: Cochrane Library, EMBASE, PubMed, Scopus and SciELO, as well as archives of national journals, were all searched for articles on STEMI in Cuba, from 2000 to March 2020. They were included if they reported number or percentage of application of reperfusion therapy; administration of aspirin, enalapril-captopril (ACEI) or beta-blockers; status of patients at discharge; and patient or system delay times. Finally, 17 reports with 7823 patients were included.Results: Thrombolytic therapy was administered to 3991 patients (51%), and 695 patients (8.9%) died. Only four studies, with 880 patients, presented data about prescription of ACEI, aspirin, and beta-blockers, which were administered to 381 (45.3%), 824 (93.6%), 464 (52.7%) patients, respectively. Coronary intervention was reported in 5 studies with 3422 patients, being performed in 661 (19.3%). Conclusions: Quality of care of patients with STEMI seems to be poorer than reported in similar scenarios. Thrombolytic administration is still low, although mortality decreases in this period. Other pharmacological treatments were insufficiently fulfilled.

## Resumen

Antecedentes: En Cuba no existe un registro nacional de Infarto Agudo de Miocardio con Elevación de Segmento ST (IAMCEST). Tampoco son publicadas métricas de calidad de atención a estos pacientes. Objetivo: Esta revisión de estudios cubanos de pacientes con IAMCEST se propone describir la calidad de la atención médica.Métodos: se localizaron artículos de IAMCEST en Librería Cochrane, EMBASE, PubMed, Scopus, SciELO y en archivos de revistas nacionales, hasta marzo de 2020. Se incluían si reportaban numero o porciento de aplicación de terapia de reperfusión, de administración de captopril/enalapril (IECA) o de betabloqueadores; estado de pacientes al egreso; demora de pacientes o Sistema. Finalmente, se incluyeron 17 estudios con 7823 pacientes. Resultados: La trombolisis fue administrada a 3991 pacientes (51%); fallecieron 695 (8.9%). Cuatro estudios con 880 pacientes presentaron información sobre prescripción de IECA, aspirina o betabloqueadores, siendo administrados a 381 (45.3%), 824 (93.6%), 464 (52.7%) pacientes, respectivamente. Cinco estudios con 3442 pacientes reportaron realización de intervencionismo coronario, practicado en 661 (19.3%). Conclusiones: La calidad de atención a pacientes con IAMCEST es más baja que en escenarios similares. La administración de trombolítico aún es deficiente, aunque la mortalidad descendió en este periodo. El resto de fármacos fueron insuficientemente administrados.

## Resumo

Fundamento: Em Cuba, não há registro de Infarto do Miocárdio com Elevação do ST (STEMI), nem análises de medidas de desempenho são amplamente divulgadas.Objetivo: Uma revisão de estudos cubanos de pacientes com STEMI foi realizada para descrever a qualidade do atendimento médico.Métodos : Biblioteca Cochrane, EMBASE, PubMed, Scopus e SciELO, bem como arquivos de revistas nacionais, foram pesquisados artigos sobre STEMI em Cuba, de 2000 a março de 2020. Eles foram incluídos se relatassem número ou porcentagem de aplicação de terapia de reperfusão ; administração de aspirina, enalapril-captopril (IECA) ou betabloqueadores; situação dos pacientes na alta; e tempos de atraso do paciente ou do sistema. Finalmente, 17 relatórios com 7.823 pacientes foram incluídos. Resultados: A terapia trombolítica foi administrada a 3.991 pacientes (51%) e 695 pacientes (8,9%) morreram. Apenas quatro estudos, com 880 pacientes, apresentaram dados sobre a prescrição de IECA, aspirina e betabloqueadores, que foram administrados a 381 (45,3%), 824 (93,6%), 464 (52,7%) pacientes, respectivamente. A intervenção coronária foi relatada em 5 estudos com 3.422 pacientes, sendo realizada em 661 (19,3%). Conclusões: A qualidade do atendimento de pacientes com STEMI parece ser pior do que o relatado em cenários semelhantes. A administração de trombolíticos ainda é baixa, embora a mortalidade diminua nesse período. Outros tratamentos farmacológicos foram insuficientemente cumpridos.

**KEY CONCEPTS**

What is known on the subject: Measuring the quality of care for patients with Acute Myocardial Infarction essentially depends on the determination of quality indicators and a registry of patients with said condition. In Cuba, there is still no National Registry of ST-segment Elevation Acute Myocardial Infarction (STEMI), and there are very few multicenter studies. In addition, the available indicators are more of an epidemiological type. Therefore, providing an estimate of the quality of care to patients with STEMI can be very difficult in these circumstances. What this work contributes: through the evaluation of reports from institutional registries of patients with STEMI, it is established that the application of reprfusion therapy is still scarce, as is compliance with the rest of the pharmacological treatment. In-hospital mortality is still high.

**Disclosure**

Most registries of patients with ST-segment elevation myocardial infarction (STEMI) from low- and middle-income countries describe a suboptimal quality of care. Despite the fact that in Cuba the minimum tools necessary for a correct description of this situation do not exist, the analysis of published reports constitutes an alternative. 17 reports of patients with STEMI were identified, with 7823 patients, of non-uniform methodology, and with heterogeneous data. It is concluded that the quality of care for patients with STEMI is still poor.

## Introduction

Acute myocardial infarction and, specifically, ST Elevation Myocardial Infarction (STEMI), represents a great burden for health professionals. ^1-3^ Despite recent advances in its treatment, reported in clinical guidelines, ^4, 5^ in low/middle income settings it can be difficult to treat a patient with acute myocardial infarction correctly. ^3, 6^

As indicated in the long-tErm follow uP of anti-thrombotic management patterns in AcutE CORonary syndrome patients (EPICOR) Registry, ^7^ Latin America was the third region with the most patients who did not receive any reperfusion strategy, (surpassed only by India and China), the third with the longest delay until coronary intervention, (again surpassed by China and India), and the one with the highest mortality at 23 months of follow-up.

In Cuba, quality metrics haven't been properly studied. There are several investigations where analyzes of performance measures were carried out, ^8, 9^ proposed metrics in national protocol only include epidemiological parameters, and cannot be uses to evaluate quality of medical attention. Also, there is no national registry of admitted STEMI patients where data can be extracted to compare centers (although there is an ongoing project for one, which will include data from 5 centers). ^10^

However, COVID-19 pandemic may negatively impact in quality of health care, including STEMI, as described. ^8, 9^ So, reports of patients with this disease in Cuba, during this pandemic, may show underestimation of real quality of attention. So, to eliminate this latter bias, we conducted a review of Cuban observational studies of STEMI patient registries prior COVID-19 pandemic to describe performance of healthcare.

## Methods

### 1- General Design of Research

This narrative review was carried out according to the preferred reporting items for systematic reviews and meta-analyses. Despite been a narrative review, inclusion in international prospective registers of systematic reviews was attempted. However, as search words included mostly terms in Spanish language, the inclusion was rejected.

Electronic databases (Cochrane Library, EMBASE, PubMed, Scopus and SciELO) were searched for articles on Acute Myocardial Infarction in Cuba published from January 2000 to August 2020, written in English or Spanish. Articles were reviewed in 3 phases: 1) by title by 2 reviewers; 2) by summary of 3 reviewers, with unresolvable disagreements between the 3 reviewers included for the next phase of screening; and 3) by full text from 4 reviewers. There were no disagreements in the final phase of screening.

Additional potential references were identified by reviewing the reference lists of included articles and subjected to the same selection process. Archives of several national journals were also reviewed, such as the Cuban Journal of Cardiology and Cardiovascular Surgery (indexed in Scopus since January 2020) and CorSalud (journal from a tertiary institution in the centern region of the country). Although, a methodology was published for the homogenization of Cuban research on Myocardial Infarction as late as 2018, the inclusion criteria were established by the authors.

Articles were included if they reported number or percentage of two of following parameters: application of reperfusion therapy; administration of aspirin, enalapril-captopril or beta-blockers; status of patients at discharge; and patient or system delay times. Articles of any study design, original articles, research letters, and editorials were eligible for inclusion (no systematic review found). Qualitative and quantitative data were extracted and organized by year, sample size and center.

Due to the heterogeneity of the data, no meta-analysis was performed. Where possible, descriptive statistics related to inclusion criteria parameters were calculated, and where only qualitative information was available, those data were abstracted. Finally, the sample per year was obtained by dividing the sample according to months of study and giving each year an integer proportional to the number of months included in each study, and the same approach was attempted for all parameters, including mortality and administration of reperfusion therapy.

### 2- Searching Methodology

Up to 127 potential articles were located in the Online Scientific Library (ScieLO) Cuba through a concise review of all the descriptors that may include reference to Acute Myocardial Infarction: "Infarto Agudo de Miocardio": 6 results; "Infarto Agudo del Miocardio": 40 results; "Infarto Agudo Miocardico": 1 result; "Infarto Cardiaco": 1 result; "Infarto de Miocardio Agudo": 13 results; "Infarto de Miocardio con Elevación del ST": 1 result; "Infarto de Miocardio": 1 result; "Infarto de Miocardio": 1 result; "Infarto del Miocardio": 57 results; "Infarto del Miocardio Agudo": 2 results; "Infarto del Miocardio con Elevación del ST": 2 results; "Infarto del Miocardio / diagnostico": 1 result; "Infarto del Miocardio / Complicacion": 1 result; "Infartos": 1 result; and "Infarto Miocardico Agudo": 1 result. A search was also carried out in regional ScieLO with the term: myocardial infarction in Cuba", and 28 overlapping results and 3 new results were obtained, but discharged due to a redundant sample.

The PUBMED search of "STEMI in Cuba" was executed and 19 results were found, but only two were included, the rest were discharged because they were not a real registry report. Likewise, with the terms Myocardial infarction in Cuba, 73 new results of discharged reports were found and they were excluded because there were no reports of hospital records.

In addition, they were excluded if they applied a sampling technique (age, sex, diagnosis, therapy, result, or any other), or were classified as a redundant sample if: another report included this same or part of this sample, making it impossible to carry out a separate analysis of the same (for this particular case, the report with the largest sample was included); and, if they included patients with non-ST elevation myocardial infarction, being impossible to carry out a separate analysis of these two diagnoses.

Our search returned a total of 78 potential citations. Figure 1 summarizes the number of articles involved in each stage of the systematic review process. At the full text selection level, most articles were excluded: 14 of them were excluded because they included other diagnoses; another 22 applied a sampling technique and 24 presented a redundant sample or only one parameter.

**Figure:**
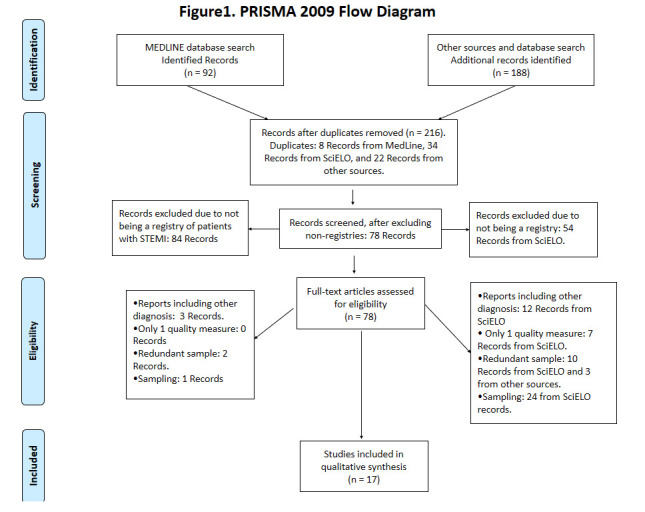

Qualitative and quantitative data were extracted and organized by year, sample, and center. Due to heterogeneity of the data, meta-analysis was not conducted. Descriptive statistics related to inclusion criteria parameters were calculated when possible. When only qualitative information was available, those data were summarized. Sample by year was obtained dividing sample according months in every year of study, and same approach was attempted for every parameter, including mortality and administration of reperfusion therapy.

The approval of the Institution's Ethics Committee was not required for this research, due to its nature. However, the principles of the Declaration of Helsinki were respected during its course.

## Results

Seventeen reports of patients with ST-segment Elevation Acute Myocardial Infarction were included in the final analysis, with 7823 patients, (10-26) during 661 months of inclusion, as shown in Table 1. Demographic characteristics were scarce and incomplete. Seven manuscripts didn't show any information about sex of patients, or presented data that didn't allow a correct determination of this parameter. Finally, only seven papers offered valid information about it, and from 5055 patients, 3410 (67.4%) were men.

According to risk factors, information was more incomplete and heterogeneous, due to lack of consensus. Some researchers may find suitable for their research to gather some information, that other may find superfluous. In case of hypertension and smoking, they were reported in 4179 patients (66.5%) and 3449 (54.9%), from a sample of 11 studies; Diabetes Mellitus was reported in 1598 patients (26.7%) from a sample of 10 studies; Ischemic heart diseases was reported in 1859 patients (34.4%) from a sample of eight studies; obesity (without reporting body mass index cut-off value) was reported only in four studies, in 962 patients (21.2%); and finally, dyslipidemia was reported in 837 patients (23.5%) from five studies.

**Table:** 

Study	Data	Data gathering method	Lang.	Patients	Sample	Mean	Male	Thromb (%)	PCI (%)	Mortality (%)
				setting	size	age	(%)			
			
Guedes-Díaz R, et al. 2009 ^11^	2005–2007	Institutional Registry	Span	Secondary center	69	64	53 (74.6)	30 (43.4)	0	15 (21.7)
Tartabull-Poutriel, et al. 2009 ^12^	2002-2006	Institutional Registry	Span	Secondary center	338	65	N/R	70 (20.7)	0	47 (13.9)
Gort-Hernandez M, et al. 2011 ^13^	2004-2010	Institutional Registry	Span	Multicenter. Primary Care	69	N/R	N/R	43 (62.3)	0	12 (17.4)
Álvarez-González L, et al. 2011 ^14^	2006-2007	Survey	Span	Tertiary center	128	N/R	N/R	50 (39.1)	34(26.6)	18 (14.1)
Achiong-Alemañy , et al. 2012 ^15^	Jun-Aug 2010	Survey	Span	Multicenter. Primary Care	88	N/R	N/R	35 (39.7)	0	8 (9.1)
Delgado Acosta HM, et al. 2013 ^16^	June 2011	Survey	Span	Secondary center	20	65	16 (80)	16 (80)	0	0
Lóriga-García O, et al. 2013 ^17^	2011-2012	Institutional Registry	Span	Secondary center	644	N/R	N/R	356 (55.2)	0	50 (7.7)
León-Aliz E, et al. 2014 ^18^	Jan 2009-Oct 2010	Institutional Registry	Span	Secondary center	128	67,3	96 (75)	53 (41.4)	35	14 (10.9)
Rodriguez-Londres J, et al. 2014 ^19^	2001-2012	Institutional Registry	Span	Tertiary center	2493	N/R	1748 (70.1)	939 (37.6)	469	177 (7.1)
Santos-Pérez A, et al. 2015 ^20^	2011	Institutional Registry	Eng	Secondary center	172	N/R	N/R	87 (50.5)	22	21 (12.2)
Betancourt-Plaza I, el al. 2016 ^21^	2014	Institutional Registry	Eng	Secondary center	115	64,7	76 (66.1)	52 (45.2)	0	12 (10.4)
Berty Gutiérrez, et al. 2017 ^22^	Jan 2015-Dec 2016	Institutional Registry	Span	Secondary center	240	N/R	N/R	85 (35.4)	N/R	17 (6)
Vitón Castillo AA, et al. 2018 ^23^	2013-2017	Institutional Registry	Span	Secondary center	1427	N/R	909 (63.7)	923 (64.7)	0	130 (9.1)
Martínez García G, et al. 2019 ^24^	Intermitent:	Institutional Registry	Span	2 Secondary centers	507	62,9	160 (31.6)	310 (61.1)	101 (19.9)	N/R
	Sept 2013-Jan 2017									
Rodriguez-Jimenez A, et al. 2019 ^25^	2014-Jun 2017	Institutional Registry	Eng	Secondary center	476	67,4	304 (63.9)	388 (81.5)	0	61 (12.8)
Santos-Medina M, et al. 2019 ^10^	Jan 2018-Apr 2019	On-line health records	Span	Secondary multicenter	791	N/R	N/R	450 (56.9)	N/R	73 (9.2)
Besse-Diaz, et al.2021 ^26^	2017-2019	Institutional Registry	Eng	2 Secondary centers	118	N/R	57 (48.3)	104 (88.1)	0	15 (12.7)

Lang.: Language. N/R: Not reported, or presented data don't allow a correct determination of this parameter

### - Analysis of measures

1. Thrombolytic administration: all 17 reports were included as they present this particular measure. A total of 3991 patients (51%) received pharmacological thrombolytic therapy.
2. In-Hospital Mortality: All 17 reports were included in this analysis. A total of 695 patients (8.9%) have been reported to have died during their in-hospital stay due to myocardial infarction.
3. Other measures: The remainder pharmacological treatment or intervention were not reported or poorly performed. Only four studies, with 880 patients, presented data on the prescription of angiotensin-converting enzyme inhibitors aspirin, and beta-blockers, which were administered to 383 (43.5%), 833 (94.7%), 464 (55.7%) patients respectively. Finally, coronary intervention was reported in 5 studies with 3428 patients, being performed in 661 patients (19.3%).

### Trend analysis

As indicated in Figure 3, the rate of thrombolytic administration increased over time and reached, in the last 6 years, a value of 57.1%, versus 46.8% in the rest of the time under study (p < 0.001). Despite what might be assumed, in-hospital mortality remained stable, when comparing these 2 study periods, and in the last 6 years, up to 8.5%. When reordering the reports by study sample, it is seen that mortality decreased meanwhile, the rate of administration of thrombolytic, the rate of reperfusion by any means, increased. Therefore, the reported analysis above may depend on the study sample, rather than a trend over time. Finally, due to the low frequency of notification of the rest of the treatment, it was not possible to perform a time trend analysis for the administration of angiotensin-converting enzyme inhibitors, aspirin, or beta-blockers.

**Figure:**
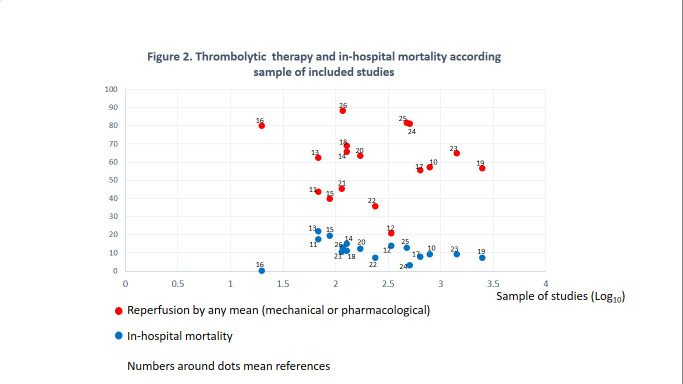

**Figure:**
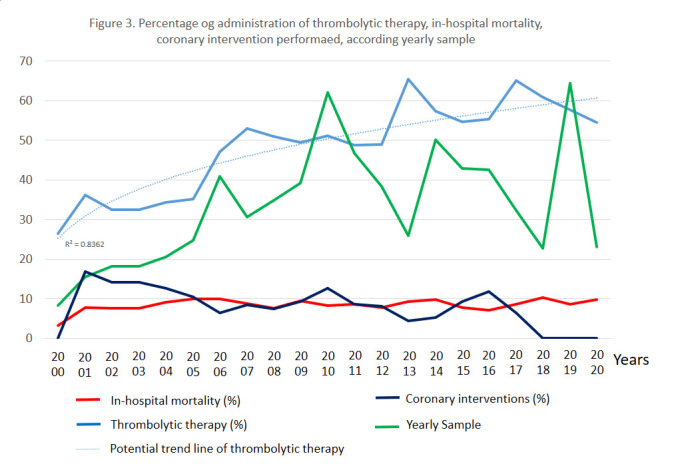

## Discussion and/or Conclusions

This narrative review attempts to describe performance of medical care in Cuban observational studies of patient admitted with STEMI. However, discussing causes of some results is beyond of the scope of this manuscript, as they may be consequences of internal policies.

Comparison with indicators of foreign registries, where patients with coronary procedures or prehospital reperfusion with thrombolytic bolus, prevail is not fair. In Cuba, only 5 centers perform coronary intervention (3 of them in Havana City). Son, in this country, suggested treatment for STEMI is pharmacological reperfusion with Heberkinase® (Recombinant Streptokinase, CIGB, The Havana, Cuba). The latter may affect the extension of these results to settings where the main treatment is interventional procedures.

However, a large number of STEMI patients, in low- and middle-income settings, are still treated with systemic thrombolytic, so there is still enough room to generalize these results. The NCDR-ACTION registry ^27^ showed that by performing more procedures, system delays were shortened and effective treatment rates were high. In addition, the use of remote procedures was lower in centers that exceeded the barrier of 400 patients / year. However, this number must be correlated with the number of personnel qualified for coronary intervention. Medium- and small-volume patient centers regulate their human resources to allow them to perform at least 34-45 procedures annually. ^28^ . However, Korea-PCI investigators ^29^ found no difference between higher-volume and lower-volume of interventions in terms of death, cardiac death, nonfatal reinfarction, or stent thrombosis. It is noteworthy that in this study, specialists with a high number of procedures only performed 28 of these per year.

### 1 Analysis of published reports

Thrombolytic administration values greater than 70% were rare, despite the fact that it is the only way to achieve reperfusion, available in the country.

A recent meta-analysis of studies of Acute Coronary Syndromes in Sub‐Saharan Africa ^30^ , reported that fibrinolysis was the most widely reported and available means of revascularization in most of included studies (18/27 or 66.7%). However, in north Cairo, after establishing a regional STEMI network, the use of primary PCI increased up to 77.1%.

According to Karthikeyan et al ^31^ , any model of STEMI care in India (a middle income country with inherent differences according provinces and even, districtes) should have the principal objectives of improving the rates of fibrinolysis, and reducing the time from symptom onset to FMC, and treatment. They think that performing ECGs at the point of FMC, enabling their prompt interpretation (by on-site or off-site personnel) and prompt initiation of bolus fibrinolytic therapy is enough to improving STEMI care in India in short term.

In Guatemala, as in Cuba, pharmacological reperfusion is the main method available given the lack of access to PCI centres. In the ACS-GT Registry, ^32^ 59.1% patients received pharmacological reperfusión. In a tertiary public hospital in a resource limiting country without coronary intervention (Trinidad), 70.5% received thrombolytic; and, in rural China, in Henan prefecture, in a multicenter study of 50 secondary hospitals with 2553 patients, fibrinolysis was administered in 52.6% of patients.

So, despite worldwide advances in coronary intervention, there is still a room to improve coverage of administration of thrombolytic, especially in low-middle income scenarios.

### 2. Mortality

In the first decade under study, a stability close to 10% is observed, as shown in Figure 3. In the second decade, a decrease in mortality is observed, which may or may not be related to the increase in pharmacological reperfusion procedures. or interventionist. However, this parameter was found to be related to the number of patients seen, as reported previously: ordering the sample of Cuban studies in increasing order, we find that the fraction of patients who died actually decreased.

These data support the hypothesis that the greater the number of patients, the higher the quality of care provided. Likewise, it coincides with several reports of analysis of mortality trends where a decrease in this parameter is reported, mainly due to the introduction of new reperfusion therapies. ^33-35^ However, in this country, no new widespread advances in STEMI reperfusion therapy have been seen since the adoption of thrombolytic therapy in the early 2000s.

In multicenter study in Henan prefecture, ^36^ in-hospital mortality was below 4% (3.9%). However, in secondary centers where coronary interventions were less performed, only rise up to 4.6%. In Trinidad^37^, the in-hospital death rate was higher (6.5%), but lower than reported in this study. In the ACS-GT Registry, ^32^ in-hospital mortality was 18.5% in STEMI patients. And in Sub-Saharan report, ^30^ this parameter was not reported in 5 studies; however, in remaining seven documents in those included only STEMI patients there was a combined in-hospital mortality of 9.3%, just quite higher than reported in this study.

### 3. Discussion of temporal analysis.

The restructuring of emergency care services between 2010 and 2011 may influence the results of this study, since they were centralized in a small number of centers. It is very likely that the number of patients with thrombolytic will decrease compared to previous years, since patients had to be transferred to units where this therapy was authorized. Note that mortality does not increase in this period, but it does 1 or 2 years later. Three years later, with the reopening of several intensive care rooms in primary care hospitals, the growing trend in the application of thrombolytic and the decrease in mortality began to be noted.

The instability of human resources could also play a causal role in the behavior of these parameters. Specialists with years of experience in diagnosis and treatment of Emergencies had to be replaced by doctors with less experience, which could result in a hypothetical delay in diagnosis and therapy. And not only human resources, but also materials (monitors, medical transport and absence of telecardiology) can currently play a determining role in accomplishing quality indicators and performance measures.

Nevertheless, the use of coronary intervention has been increasing over time. And although there are few centers with the capacity to do it in this country, the number of patients has increased. In the Cuban Registry of Acute Myocardial Infarction, ^10^ a low rate of application of interventionism was found, barely 3.6%. the low performance rate of coronary intervention in this study was due to that most of the patients in participating institutions did not come from PCI-capable canters.

In Argentina ^38^ , in a report of patients with coronary interventions, less than 60% of patients received ACE inhibitors or beta-blockers. In Brazil, 47.5% of the patients in the subgroup treated in the Public Health System of VICTIM (Vía Crucis para el Treatment del Infarto de Miocardio) ^(39)^ received reperfusion therapy, but only 2 of them received a pharmacological approach. Double antiplatelet therapy was administered to more than 97% of the patients, however ACEI and beta-blockers, to only 73% and 31%, respectively.

In Mexico, in RENASCA patients ^33^ , aspirin was administered at 88.6%, however, beta-blockers and ACE inhibitors did not reach 40%. In this report, which specifically describes changes in performance measures before and after implementation of the "Infarct Code", patients without any reperfusion decreased from 65% to 29%. Patients with thrombolytics increased from 25.5% to 40%. Due to the paucity of data from lower-middle income settings, there is no way to establish a trend in this region to compare the results of this report. And, as noted above, comparison with data from high-income or other settings would not be fair to trends in this low/middle income country without coronary intervention.
